# The Efficacy of Tranexamic Acid in Pediatric Tonsillectomy: A Systematic Review and Meta-Analysis

**DOI:** 10.7759/cureus.92834

**Published:** 2025-09-21

**Authors:** Nawaf Almotairi, Nawaf Alghamdi, Raghad Alghamdi, Alwaleed Alamri, Ahmed Alshehri, Khaled Alshehri, Turki Alzubaidi, Ghada A Bin Abbas, Rawan Altalhi, Reema Albalawi, Rimah Alsalem, Shahad Almalki, Mariam AlRouqi, Marwan Al-Qunaee

**Affiliations:** 1 Otolaryngology - Head and Neck Surgery, Zain Hospital, Kuwait City, KWT; 2 Otolaryngology - Head and Neck Surgery, King Abdulaziz University Faculty of Medicine, Jeddah, SAU; 3 Otolaryngology - Head and Neck Surgery, Al-Baha University, Al-Baha, SAU; 4 Otolaryngology - Head and Neck Surgery, King Khaled University, Abha, SAU; 5 Otolaryngology - Head and Neck Surgery, Taif University, Taif, SAU; 6 Otolaryngology - Head and Neck Surgery, University of Tabuk, Tabuk, SAU; 7 Otolaryngology - Head and Neck Surgery, King Saud University Medical City, Riyadh, SAU; 8 Otolaryngology - Head and Neck Surgery, King Fahad Medical City, Riyadh, SAU

**Keywords:** bleeding risk, blood loss, efficacy of tranexamic acid, pediatric, perioperative tranexamic acid, post-tonsillectomy hemorrhage, tonsillectomy, tranexamic acid uses

## Abstract

Complications of tonsillectomy, such as intraoperative and post-tonsillectomy hemorrhage (PTH), are not uncommon. In children, these may lead to hypovolemic shock, airway obstruction, delayed healing, and infection, among other issues. Tranexamic acid (TXA) is one of several hemostatic agents. However, its efficacy in reducing bleeding during tonsillectomy is not well known. Hence, we aimed to determine whether children undergoing tonsillectomy experienced less perioperative hemorrhage after receiving a preoperative dose of TXA. Two reviewers independently conducted a systematic search of three databases (PubMed, Google Scholar, and the Cochrane Central Register of Controlled Trials [CENTRAL]) until June 2024. This review included all randomized controlled trials (RCTs) involving pediatric patients (<18 years) undergoing tonsillectomy, focusing on studies that evaluated the efficacy of preoperative TXA compared with a control group in reducing intraoperative or postoperative bleeding. The studies varied in outcomes, designs, and sample sizes. A meta-analysis of four studies (n = 380) revealed no significant effect of TXA in reducing intraoperative bleeding (weighted mean difference = -54.44 (95% confidence interval [CI] = -110.98 to -2.10, p= 0.06). Preoperative TXA did not significantly reduce intraoperative bleeding. Future RCTs should assess surgical techniques, TXA dosage, bleeding measurement, patient age, and postoperative medications.

## Introduction and background

Tonsillectomy is a common pediatric procedure primarily performed to treat obstructive sleep apnea (OSA) and recurrent tonsillitis [1]. OSA affects approximately 0.7% of children aged four to five years, with symptoms including chronic mouth breathing, snoring, restless sleep, and frequent awakenings during the night [2]. Otolaryngologists must be mindful of the complications associated with tonsillectomy, such as vomiting, nausea, postoperative pain, dehydration, airway obstruction, and pulmonary edema, with bleeding both intraoperatively and postoperatively being among the most frequent [3,4] and potentially life-threatening complications [5]. According to clinical practice guidelines for pediatric tonsillectomy, the predominant causes of hospital readmission after the procedure are primary and secondary hemorrhage [3]. Therefore, strategies to prevent these complications are crucial.

The management of post-tonsillectomy hemorrhage (PTH) ranges from simple observation to more severe interventions, including cauterization in the operating room under general anesthesia [6]. Antifibrinolytics have demonstrated effectiveness in reducing blood loss in different settings, including cardiac surgery, trauma, liver surgery, solid organ transplantation, and certain non-surgical procedures [7]. These agents may reduce the necessity for general anesthesia and surgical procedures [8]. However, their efficacy in reducing PTH remains controversial, possibly due to inconsistent evidence across studies, variations in surgical techniques, and patient-related differences. Due to the limited research evidence that validates the efficacy of tranexamic acid (TXA) in reducing bleeding during tonsillectomy, its use in such surgery remains inconclusive. The goal of this meta-analysis and systematic review was to determine whether children undergoing tonsillectomy experienced less perioperative hemorrhage after receiving a preoperative dose of TXA.

## Review

Materials and methods

Objective and Search Strategy

The primary goal was to evaluate randomized controlled trials (RCTs) that assessed the effectiveness of preoperative TXA in reducing perioperative bleeding during juvenile tonsillectomy. This meta-analysis has a PROSPERO registration number (CRD42024547418) and adhered to the Preferred Reporting Items for Systematic Reviews and Meta-Analysis (PRISMA) standards [9]. An extensive electronic search was performed on three databases (PubMed, Google Scholar, and the Cochrane Central Register of Controlled Trials [CENTRAL]), encompassing research from the beginning of the databases until June 2024. The search parameters were chosen to assess existing published literature that discusses the results of TXA in pediatric tonsillectomy, specifically focusing on its impact on intra-operative blood loss, PTH rates, associated adverse effects, the need for further surgical interventions, and postoperative recovery quality, as assessed in RCTs. Keywords such as "pediatric tonsillectomy," "tranexamic acid," "TXA," "tonsil surgery," "childhood tonsillar surgery," "postoperative hemorrhage," "intraoperative blood loss," "surgical bleeding," and "prophylactic tranexamic acid" were used in the search. To improve the search, Boolean operators (AND, OR) were utilized.

Study Selection Criteria

In this review, we exclusively considered articles written in English and included only full-length RCTs involving pediatric patients (<18 years) undergoing tonsillectomy and studies assessing the efficacy of preoperative TXA compared with a control group (placebo or no intervention). Eligible studies were required to report outcomes such as intraoperative blood loss, PTH rates, and any related adverse effects. No minimum follow-up duration was required for inclusion. Exclusion criteria were as follows: studies involving adult or mixed populations without separate data for pediatric patients, non-RCTs, observational studies, case reports, reviews, and studies that did not report the specified outcomes. Studies involving adenotonsillectomy were also excluded unless they provided data isolating the effect of TXA on tonsillectomy alone.

Article Selection and Data Extraction

Two reviewers independently conducted systematic searches for papers meeting these criteria. Titles and abstracts were initially screened for relevance, followed by full-text reviews of potentially eligible articles. The selected publications were methodically examined for study design, participant demographics, intervention specifics, and outcome measures. Discrepancies between reviewers were resolved through consultation with a third reviewer. Each included RCT was assessed for bias using the Cochrane Risk of Bias tool (RoB 2) by two independent reviewers.

Statistical Analysis

We conducted a meta-analysis using Review Manager (RevMan) to systematically analyze data from multiple RCTs. We defined statistical significance as p<0.05.

Results

Literature Search

A total of 195 publications were identified through a comprehensive search, including 26 from PubMed, 166 from Google Scholar, and three from Cochrane Central. After screening titles and abstracts, 187 articles were excluded. The remaining full-text articles were assessed for eligibility. Five RCT studies were included in the final analysis.

Characteristics of Included Studies

The included studies varied in design, sample size, and measured outcomes. Sample sizes ranged from 16 to 100 participants per study, with mean participant age ranging from four to 15 years. Some studies reported specific age ranges, such as 6-10 years (Table 1). TXA was administered preoperatively in various forms (oral, topical, or intravenous) at doses of either 15 mg/kg or 20 mg/kg and compared with placebo or no intervention. Follow-up durations were not extensively detailed, with only one study reporting a 1-month follow-up period. The studies were conducted in various countries: one in Saudi Arabia, two in Egypt, one in Pakistan, and one in India (Table 1).

**Table 1:** Descriptive summary of included studies TXA: tranexamic acid; SD: standard deviation

Studyy	Year of publication	Country	Sample size (total)	Sample size (TXA group)	Sample size (control group)	Age, years, mean ± SD	Sex distribution (male, n: %, female, n: %)
Aboelsuod et al. 2023 [10]	2023	Egypt	82	41	41	Group T: 7.71 ± 2.59; Group N: 7.33±2.13	Group T: 23: 56.1%, 18: 43.9%; Group N: 25: 61%, 16: 39%
Elgebaly and Elzayat, 2018 [11]	2018	Egypt	100	50 (Group 2)	50 (Group 1)	Group 1: 9.2 ± 1.62; Group 2: 8.3 ± 1.85	Group 1: 28: 56%, 22: 44%; Group 2: 30: 60%, 20: 40%
Soliman and Alshehri, 2015 [12]	2015	Saudi Arabia	225	150 (Group A, 75; Group B, 75)	Group C, 75	Group A: 7.14 ± 2.35; Group B: 7.00 ± 2.32; Group C: 7.10 ± 2.45	111: 49.3%, 114: 51.8%
Ashraf et al. 2022 [13]	2022	Pakistan	27	13	14	Not specified	Not specified
Santosh, 2016 [14]	2016	India	37	19	18	Not specified	Not specified

**Figure 1:**
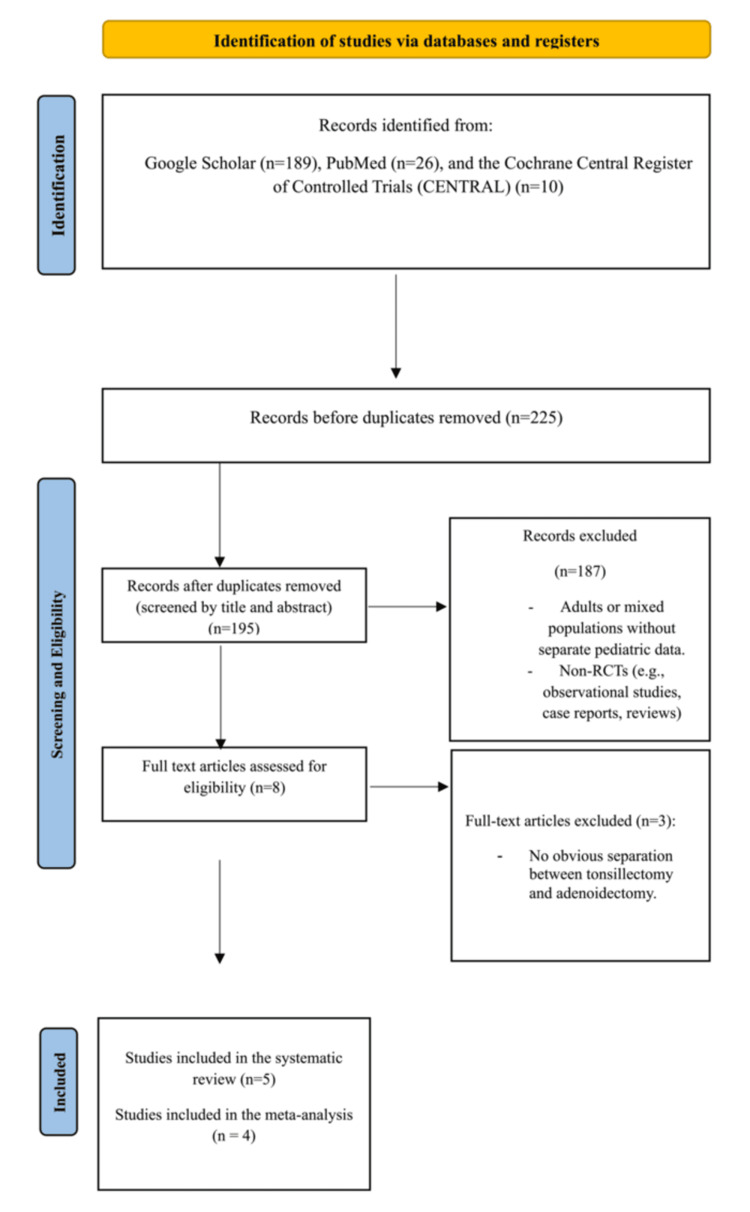
PRISMA flowchart depicting the selection of studies PRISMA: Preferred Reporting Items for Systematic Reviews and Meta-Analysis

Collectively, these studies provided valuable insights into the safety and efficacy of TXA in minimizing perioperative bleeding in pediatric patients undergoing tonsillectomy (Figure 1).

Risk of Bias Assessment

Using the RoB 2 tool, we assessed the risk of bias in the eligible RCTs (Table 2). Three studies were found to have a high risk of bias, while two exhibited moderate risk [10,11]. The two major sources of bias were related to outcome assessment and insufficient blinding of participants and personnel.

**Table 2:** Risk of bias in reviewed studies

Study	Selection bias	Performance bias	Detection bias	Attrition bias	Reporting bias	Other bias	Overall bias
Aboelsuod et al. 2023 [10]	Low	Low	Low	Low	Low	None	Low
Elgebaly and Elzayat, 2018 [11]	Low	Low	Low	Low	Low	None	Low
Soliman and Alshehri 2015 [12]	Low	Unclear	Unclear	Low	Low	None	High
Ashraf et al. 2022 [13]	High	Unclear	Unclear	Low	Low	None	High
Santosh, 2016 [14]	Low	Unclear	Unclear	Low	Low	None	High

Intraoperative Blood Loss

**Figure 2:**
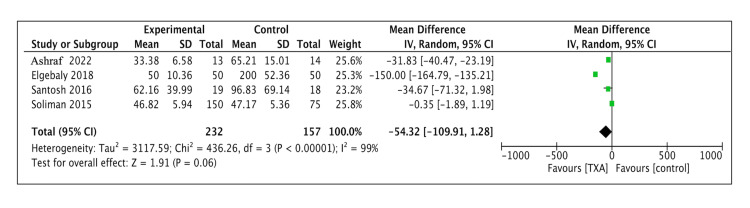
Forest plot diagram demonstrating the effect of tranexamic acid on intraoperative blood loss CI: confidence interval; SD: standard deviation

Four studies (n = 380: 230 in the TXA group and 150 in the control group) reported detailed data on intraoperative blood loss. The meta-analysis found no significant effect of TXA for reducing intraoperative bleeding, with a weighted mean difference of -54.44 (95% confidence interval [CI] = -110.98 to -2.10, p = 0.06) (Figure 2).

Discussion 

We identified five clinical trials to test the hypothesis that preoperative use of TXA in pediatric patients undergoing tonsillectomy would reduce intraoperative and postoperative bleeding compared with placebo or no interventions [10-14]. A meta-analysis of four studies found no statistically significant difference between the groups (p = 0.06) [11-14]. Soliman and Alshehri examined the effect of administering TXA to 150 pediatric patients compared with 75 controls and found no significant differences in blood loss or complications between the groups [12]. In contrast, in an RCT conducted by Ashraf et al., a significant difference in blood loss was observed in the pediatric population aged 4-14 years between the TXA group and the no intervention group (p<0.001) [13]. Similar findings were reported by Elgebaly and Elzayat, who found a highly significant difference in blood loss between the group receiving TXA and the group with no intervention (n = 100, p<001) [11]. Two additional clinical studies by Santosh and Aboelsuod et al. further support these findings, indicating that the preoperative use of TXA significantly decreased intraoperative and postoperative blood loss during tonsillectomy in both adult and pediatric populations [10,14].

In a study by Achakzai et al., 100 patients aged 10-30 years were assigned to two groups: one receiving preoperative TXA, and the other receiving normal saline. The TXA group experienced significantly less intraoperative blood loss and shorter operative times compared with the normal saline group (p<05) [15]. Conversely, George et al. did not find a reduction in operative time between the TXA group and the placebo group in their study of 100 patients undergoing tonsillectomy. However, they did report a statistically significant reduction in intraoperative blood loss in the TXA group, with no side effects observed in either adult or pediatric patients [16].

A recent systematic review and meta-analysis conducted by Kuo et al. evaluated the prophylactic role of TXA in tonsillectomy across 10 prospective and retrospective studies involving 111,898 adult and pediatric patients [17]. Their study showed no significant reduction in intraoperative blood loss (p = 0.16) but a significant decrease in the rate of PTH (p<0.0001). However, no significant difference was observed regarding additional interventions, such as blood transfusions, the use of ethamsylate, or postoperative general anesthesia to achieve hemostasis.

Our study is distinct in its exclusive focus on the pediatric population and the inclusion of RCTs only, which provide a higher level of evidence regarding the efficacy of TXA in minimizing blood loss during pediatric tonsillectomy. Since tonsillectomies are more common in children than in adults, this pediatric-specific approach strengthens the clinical relevance of our findings. However, the study is limited by the inclusion of only four RCTs in the meta-analysis. Further large-scale, high-quality RCTs are needed to better understand the effect of TXA on peri-tonsillectomy blood loss and to assess its safety in pediatric patients.

## Conclusions

The findings of our meta-analysis showed that preoperative TXA administration does not significantly reduce intraoperative blood loss in pediatric patients undergoing tonsillectomy. Future RCTs should standardize surgical techniques, TXA dosage, and methods of measuring blood loss to address the heterogeneity observed across studies. Additionally, factors such as patient age and postoperative medications should be considered, as they may influence hemorrhage rates. Due to insufficient data on PTH across studies, a meta-analysis of this outcome could not be conducted.

## References

[1] Ong AA, Gillespie MB (2024). Tonsillectomy: contemporary indications, techniques, and outcomes. Otolaryngol Clin North Am.

[2] Locci C, Puci MV, Saderi L, Sotgiu G, Zanza C, Antonucci R (2024). The complex link between sleep-disordered breathing and asthma control in pediatric patients: A cross-sectional study. Respir Med.

[3] Mitchell RB, Archer SM, Ishman SL (2019). Clinical practice guideline: tonsillectomy in children (update)-executive summary. Otolaryngol Head Neck Surg.

[4] Harounian JA, Schaefer E, Schubart J, Carr MM (2016). Pediatric adenotonsillectomy and postoperative hemorrhage: demographic and geographic variation in the US. Int J Pediatr Otorhinolaryngol.

[5] Maksimoski M, McCauley M, Osoba M, Pirotte M, Liddy W (2024). Treatment of post-tonsillectomy hemorrhage with nebulized tranexamic acid: initial investigation of a novel therapeutic modality. Ann Otol Rhinol Laryngol.

[6] Spencer R, Newby M, Hickman W, Williams N, Kellermeyer B (2022). Efficacy of tranexamic acid (TXA) for post-tonsillectomy hemorrhage. Am J Otolaryngol.

[7] Ng W, Jerath A, Wąsowicz M (2015). Tranexamic acid: a clinical review. Anaesthesiol Intensive Ther.

[8] Abtahi M, Kargoshai AA, Shetabi H, Manafi A, Manafi N, Badrouj A (2023). The effect of tranexamic acid local injection on bleeding during and after tonsillectomy: a double-blind randomized placebo-controlled trial. World J Plast Surg.

[9] Page MJ, McKenzie JE, Bossuyt PM (2021). The PRISMA 2020 statement: an updated guideline for reporting systematic reviews. BMJ.

[10] Aboelsuod MAA, Abdalla AM, Ahmed IMA, Seyam SH, Hassan AM (2023). Clinical efficacy of local infiltration of lidocaine and tranexamic acid application in tonsillar region on postoperative pain and bleeding during tonsillectomy: prospective, randomized, double-blind controlled study. Ain-Shams J Anesthesiol.

[11] Elgebaly A, Elzayat SD (2018). The role of tranexamic acid in improving quality of pediatric tonsillectomy: a double-blinded randomized controlled study. Menoufia Med J.

[12] Soliman R, Alshehri A (2015). Assessment of the effect of tranexamic acid on perioperative bleeding in pediatric patients undergoing tonsillectomy. Egypt J Anaesth.

[13] Ashraf MA, Ahmed I, Bhatti S, Rai D, Tasleem RF (2022). Compare mean blood loss in patients undergoing tonsillectomy with and without tranexamic acid. Pak J Med Health Sci.

[14] Santosh U (2016). A comparative study to verify the efficacy of preoperative intravenous tranexamic acid in control of tonsillectomy bleeding: a comparative study to verify the efficacy of preoperative intravenous tranexamic acid in control of tonsillectomy bleeding. Otorhinolaryngol Clin.

[15] Achakzai A, Achakzai MA, Achakzai HU (2020). Efficacy of tranexamic acid in reducing intra-operative bleeding during tonsillectomy. Int J Front Sci.

[16] George A, Kumar R, Kumar S, Shetty S (2011). A randomized control trial to verify the efficacy of pre-operative intra venous tranexamic acid in the control of tonsillectomy bleeding. Indian J Otolaryngol Head Neck Surg.

[17] Kuo CC, DeGiovanni JC, Carr MM (2022). The efficacy of tranexamic acid administration in patients undergoing tonsillectomy: an updated meta-analysis. Ann Otol Rhinol Laryngol.

